# A Comprehensive Phylogeny Reveals Functional Conservation of the UV-B Photoreceptor UVR8 from Green Algae to Higher Plants

**DOI:** 10.3389/fpls.2016.01698

**Published:** 2016-11-15

**Authors:** María B. Fernández, Vanesa Tossi, Lorenzo Lamattina, Raúl Cassia

**Affiliations:** Instituto de Investigaciones Biológicas, Facultad de Ciencias Exactas y Naturales, Universidad Nacional de Mar del Plata–Consejo Nacional de Investigaciones Científicas y TécnicasMar del Plata, Argentina

**Keywords:** UVR8, UV-B, plants, phylogenetic analysis, evolutionary conservation

## Abstract

Ultraviolet-B (UV-B) is present in sunlight (280–315 nm) and has diverse effects on living organisms. Low fluence rate of exposure induces a specific photomorphogenic response regulated by the UV-B response locus 8 (UVR8) receptor. UVR8 was first described in *Arabidopsis thaliana.* In the absence of stimuli it is located in the cytoplasm as a homodimer. However, upon UV-B irradiation, it switches to a monomer and interacts with the ubiquitin ligase E3 COP1 via the UVR8 β-propeller domain and the VP core. This induces the expression of the transcription factor HY5 leading to changes in the expression of genes associated with UV-B acclimation and stress tolerance. UVR8 senses UV-B through tryptophan residues being Trp233 and 285 the most important. Based on the comparison and analysis of UVR8 functionally important motifs, we report a comprehensive phylogeny of UVR8, trying to identify UVR8 homologs and the ancestral organism where this gene could be originated. Results obtained showed that *Chlorophytes* are the first organisms from the *Viridiplantae* group where UVR8 appears. UVR8 is present in green algae, bryophytes, lycophytes, and angiosperms. All the sequences identified contain tryptophans 233 and 285, arginines involved in homodimerization and the VP domain suggesting they are true UVR8 photoreceptors. We also determined that some species from bryophytes and angiosperms contain more than one UVR8 gene copy posing the question if UVR8 could constitute a gene family in these species. In conclusion, we described the functional conservation among UVR8 proteins from green algae to higher plants.

## UVR8 Mechanism of Action and Evolutionary Conservation

Ultraviolet-B (UV-B) radiation is present in sunlight (280–315 nm). High doses of UV-B may damage macromolecules, including DNA, and induce the production of reactive oxygen species (ROS), affecting cell integrity and viability (Jordan, 1996; Brosché and Strid, 2003; Frohnmeyer and Staiger, 2003).

Since UV-B penetration in the water column is lower than in terrestrial environments (Rozema et al., 2002), a mechanism to avoid UV-B damage has evolved during the transition of aquatic to land plant. As UV-B was increasing, harboring a potential damage to DNA and photosystem II, a UV-B receptor was necessary to command defense responses for the protection of photosynthetic organisms (Tilbrook et al., 2013).

The levels of UV radiation on the Archean Earth were several orders of magnitude higher than the current level (Cnossen et al., 2007). Ancient photosynthetic organisms like cyanobacteria and various eukaryotic algae, including some green alga members, had mycosporine-like amino acids (MAAs) which are UV-B protectors (Rozema et al., 2002; Llewellyn and Airs, 2010; Rastogi and Incharoensakdi, 2013). Land plants could co-evolve with ambient UV-B levels through the evolution of UV-B absorbing polyphenolic compounds which increased in complexity from algae to higher plants (Rozema et al., 2002).

Low UV-B fluence is a signaling stimulus that regulates various metabolic and developmental processes and induces a photomorphogenic response regulated by the specific UV-B receptor UV-B response locus 8 (UVR8). UVR8 was first reported in *Arabidopsis thaliana* (Favory et al., 2009; Rizzini et al., 2011; Christie et al., 2012; Heijde and Ulm, 2012; Wu et al., 2012; Huang et al., 2014).

In the absence of stimuli, UVR8 is located in the cytoplasm as a homodimer. After UV-B irradiation, UVR8 changes to monomeric form and interacts with the ubiquitin ligase E3 COP1, avoiding the degradation of the transcription factor Elongated Hypocotyl 5 (HY5). HY5 up-regulates the expression of genes associated with UV-B acclimation and stress tolerance (Heijde and Ulm, 2012). Furthermore, two of these genes are the proteins Repressor of UV-B photomorphogenesis 1 and 2 (RUP1 and RUP2). When UVR8 interacts with RUP1 and RUP, it switches from monomer to dimer, leading to UVR8 inactivation (Heijde and Ulm, 2012). For a review see (Ulm and Jenkins, 2015).

Some evolutionary reconstructions of the UVR8 phylogeny have been reported, but they used the UVR8 putative sequences from few species (Wu et al., 2012; Tilbrook et al., 2013). Here, we report a more comprehensive phylogeny of UVR8, trying to identify the ancestral organism where this gene could be originated. We also analyze the presence of UVR8 functionally important motifs to identify UVR8 putative homologs.

The *At*UVR8 protein primary sequence (AAD43920.1) was used as template to perform a PSI-BLASTp against the *Viridiplantae* database from NCBI. Sequences retrieved were aligned with MAFFT^1^ and selection of phylogenetic informative regions from the multiple sequence alignment was performed using the BMGE 1.12 software (Criscuolo and Gribaldo, 2010). Finally, the phylogenic tree was performed using PHYML 3.0 software (Guindon et al., 2010).

**FIGURE 1:**
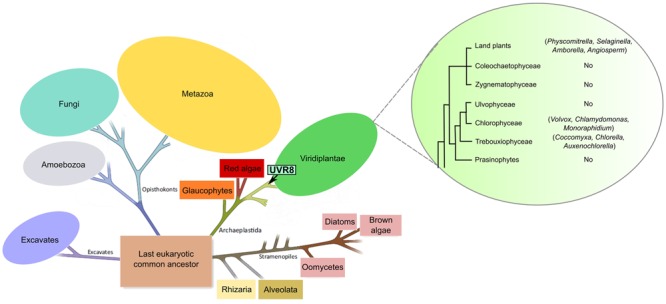
**Distribution of *At*UVR8 homologs in the Tree of Life.** Schematic representation of the eukaryotic tree of life and the *Viridiplantae* group illustrating the evolution of the UVR8 gene. The arrow indicates the origin of the UVR8 photoreceptor in the *Viridiplantae* clade. Taxons where this protein was identified are indicated in parenthesis after the name of each group. The image has been taken and adapted from Jeandroz et al. (2016) and Rensing (2016).

**Figure 1** shows the UVR8 phylogenetic tree (for a detailed phylogenetic reconstruction using the maximum likelihood method Supplementary Figure S1). Results obtained show that *Chlorophytes* UVR8 are the earliest branching members from the *Viridiplantae* group with a strongly supported clade (76.6% bootstrap support) containing the freshwater unicellular species *Auxenochlorella protothecoides, Coccomyxa subellipsoidea* C-169, *Chlorella variabilis, Monoraphidium neglectum*, and *Chlamydomonas reinhardtii*, and the multicellular species *Volvox carteri f. nagariensis* (**Figure 1**; Supplementary Figure S1). UVR8 homologs were also found in the moss *Physcomitrella patens* (*Bryophyte)* and in the seedless vascular plant *Selaginella moellendorffii* (*Lycophyte*) (**Figure 1**; Supplementary Figure S1). In seed plants, UVR8 homologs are widely present with a clear separation between monocots and dicots as shown in the maximum likelihood inferred tree (Supplementary Figure S1).

A UVR8 homolog was not found for Gymnosperm, neither in the *Viridiplantae* database nor in individual partial sequences of the genus *Ginkgo, Cycas, Zama, Chamaecyparis, Cryptomeria, Taiwania, Gnetum, Welwitschia, and Pinus*. This might be due to the absence of whole genome sequences for gymnosperm species.

### Critical Amino Acids Responsible of UV-B Perception

Ultraviolet-B resistance 8 is the first photoreceptor described who does not sense light using a prosthetic chromophore. Instead, UV-B perception in UVR8 is mediated by tryptophan residues (O’Hara and Jenkins, 2012; Ulm and Jenkins, 2015).

**FIGURE 2:**
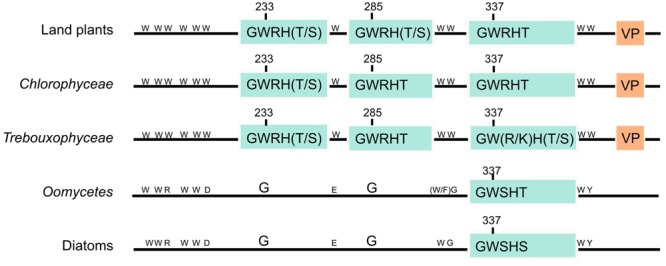
**Structural organization of *At*UVR8 homologs.** The schematic domain structure organization of *Arabidopsis thaliana* UVR8 homologs is represented for land plants, *Chlorophyceae* and *Trebouxiophyceae* taxons. Groups with proteins showing an identity lower than 40% to *At*UVR8 and containing six/seven tryptophans, corresponding to *Oomycetes* and diatoms, are also shown. Light blue boxes represent the three “GWRHT” motifs containing tryptophans 233, 285, and 337 involved in UV-B perception; orange boxes represent the “VP” domain which contributes to UVR8–COP1 interaction. The fourteen tryptophan amino acids characteristic from *At*UVR8 and substitutions are indicated in the amino acid one letter code.

*At*UVR8 has 14 tryptophan residues. Each UVR8 monomer contains the conserved pentapeptide repeat Gly-Trp-Arg-His-Thr (GWRHT) in blades 5, 6, and 7. This motif generates a triad of closely packed tryptophans (W233, W285, and W337) which are key for UV-B photoreception, W285 being the main UV-B sensor (Christie et al., 2012; Wu et al., 2012; Zeng et al., 2015). W233 is also important, both in photoreception and in maintaining exciton coupling, whereas W337 plays an auxiliary role (Christie et al., 2012; Wu et al., 2012). The “GWRHT” motif from blade 6 may be the most important because it contains W285. Supplementary Figure S2 shows that this motif is conserved in all UVR8 homologs analyzed, except for one copy of the UVR8 genes from *Medicago truncatula*. Moreover, several dicotyledonous as *Glycine max, Glycine soja, Vigna angularis, Phaseolus vulgaris, Medicago truncatula* and *Cicer arietinum* have a conservative missense mutation of threonine by serine (**Figure 2**; Supplementary Figure S2). In addition, the same mutation has been observed in the “GWRHT” motif from blade 5 in the chlorophytes *Coccomyxa subellipsoidea* C-169, *Volvox carteri f. nagariensis, Chlamydomonas reinhardtii, Chlorella variabilis* and *Monoraphidium neglectum* (**Figure 2**; Supplementary Figure S2). The “GWRHT” motif from blade 7 is conserved in all the species analyzed, except for *Phoenix dactylifera* and *Medicago truncatula* (**Figure 2**; Supplementary Figure S2). Particularly, *Chlorella variabilis* has a conservative arginine for lysine substitution and *Auxenochlorella protothecoides* a mutation of the threonine for a serine in the same motif (Supplementary Figure S2).

Conservation of the “GWRHT” motifs and tryptophan residues among UVR8 homologs reveal that most of the proteins identified in this work are true UVR8 photoreceptors. To verify it, we analyzed other UVR8 properties as the presence of the C27 domain (involved in UVR8–COP1 interaction) and the predicted UVR8 homodimerization.

### The “VP” Domain: Key Amino Acids in UVR8–COP1 Interaction

Ultraviolet-B dependent interaction of UVR8 with COP1 is a key event in UV-B signaling (Heijde and Ulm, 2012; Liu et al., 2013; Jenkins, 2014). This interaction occurs in two ways: (1) in a UV-B dependent manner via the UVR8 β-propeller domain of UVR8 with WD40 repeats domain of COP1 and (2) in a constitutively UV-B independent way via the “VP” (Val-Pro) core present in the UVR8 C27 domain (in *A. thaliana* residues 397–423) (Cloix et al., 2012; Yin et al., 2015). In contrast with COP1, the WD40 repeat proteins RUP1 and RUP2 interact with UVR8 only by the C27 domain (Yin et al., 2015).

We analyzed the presence of the C27 domain in *A. thaliana* UVR8 homologs. **Figure 2** and Supplementary Figure S2 show that although C27 domain was not well conserved, the “VP” core was conserved in 97 of 102 plant sequences (95)%. VP was absent only in the green algae *Monoraphidium neglectum, Chlamydomonas reinhardtii* and *Volvox carteri f. nagariensis*, in the angiosperms *Medicago truncatula* and *Phoenix dactylifera* and in the human RCC1 protein (Supplementary Figure S2). These results confirm that most of the proteins analyzed in this work might interact with COP1, leading to an UV-B response.

Rizzini et al. (2011) reported a *Chlamydomonas reinhardtii* UVR8 sequence lacking the C-terminal region that included the C27 domain. However, Tilbrook et al. (2016) recently described the presence of a full length UVR8 homolog in *C. reinhardtii*, suggesting that the former sequence was incomplete. *Volvox carteri* UVR8 described by Rizzini et al. (2011), also lacked the C27-including C-terminal region. However, our study revealed a full length *Vc*UVR8 protein, indicating erroneous annotation in several chlorophytes genomes.

### UVR8 Homodimerization

*At*UVR8 dimer integrity is maintained by electrostatic interactions between charged amino acids across the interaction surface, being arginine, glutamate, and aspartate especially important (Christie et al., 2012; Wu et al., 2012). Mutations in R286 and R338 produce constitutive UVR8 monomers, indicating a central role for these amino acids in maintaining the homodimeric state (Wu et al., 2012). R286 from blade 6 and R338 from blade 7 of one UVR8 molecule interact with D96 and D107 from blade 2 and D44 and E43 from blade 1 of the other molecule, respectively (Christie et al., 2012; Wu et al., 2012).

We analyzed the presence of these residues in the UVR8 homologs. Supplementary Figure S2 shows that 99% of the sequences identified (101 from a total of 102 plant sequences) contain the residue R286 and 98% the residues D96and D107 (100 sequences from a total of 102). Also, 97% of the sequences identified (99 from a total of 102 plant sequences) contain the R338, 96% (98 from a total of 102) D44 and 95% (97 from a total of 102) E43, respectively. The presence of these crucial residues in most of the proteins identified in this work suggests their ability to form homodimers in the absence of UV-B stimulus. R286 and 338 are absent in *Medicago truncatula* (XP_013442749.1) and D44, D96, D107, and E43 were not found in *Spinacia oleracea* and *Triticum Urartu* (Supplementary Figure S1), suggesting that any of these homologs’ might be able to form homodimers, being present as constitutive monomers. That poses the question of the existence of constitutive functional UVR8 monomers.

The conservation of the “GWRHT” motifs, the “VP” core and the amino acids involved in dimer integrity reported in this work, suggest the existence of functional *At*UVR8 homologs from green algae to higher plants. *At*UVR8 induces the expression of genes of the phenylpropanoid pathway as chalcone synthase (CHS; Kliebenstein et al., 2002). A phylogenetic analysis detected CHS in the green algae *C. reinhardtii*, the moss *P. patens*, the lycophyte *S. moellendorffii* and several higher plants (Wolf et al., 2010). Thus, the presence of UVR8 and CHS in these species shows a clear conservation of the UV-B signaling pathway in plants.

Recent studies have reported the cloning and functional characterization of UVR8 orthologs in *Chlamydomonas reinhardtii, Malus domestica, Populus euphratica*, and *Vitis vinicola*, these proteins being the same as those identified in this work (Liu et al., 2015; Mao et al., 2015; Tilbrook et al., 2016; Zhao et al., 2016). This finding reinforces the power of phylogenetic studies in the identification of true homologs. In accordance with conservation of key amino acids and domains described above for these proteins (Supplementary Figure S1), they have functional similarities with *At*UVR8. UVR8 expression is constitutive in *A. thaliana* (Kliebenstein et al., 2002; Kaiserli and Jenkins, 2007; Favory et al., 2009), *Vitis vinicola* (Liu et al., 2015), *Populus euphratica*, (Mao et al., 2015), and *Malus domestica* (Zhao et al., 2016). Heterologous expression of *M. domestica* or *P. euphratica* UVR8 in *Arabidopsis uvr8* mutant under UV-B irradiation showed that both proteins are able to regulate hypocotyl elongation and gene expression controlling the photomorphogenic response (Mao et al., 2015; Zhao et al., 2016). Moreover, the recently described UVR8 ortholog from *C. reinhardtii*, shows conservation in tryptophans residues critical for UV-B perception, monomerizes upon UV-B exposure, interacts with *Cr*COP1, and complements the *Arabidopsis uvr8* mutant (Tilbrook et al., 2016).

All these results are clear evidence of the conserved structure-function relationship of the UV-B receptor in plants.

## Does UVR8 Constitute a Gene Family in Some Species?

The photoreceptor phytochromes, cryptochromes, phototropins, and zeitlupe are encoded by gene families (Ahmad et al., 1998; Sharrock, 2008; Abdurakhmonov et al., 2010; Kami et al., 2010; Chaves et al., 2011). In contrast, UVR8 has been described as a single copy gene in *A. thaliana* (Brown et al., 2005). Moreover, the knock out mutant *uvr8* has null response to UV-B radiation (Favory et al., 2009). As shown in Supplementary Figure S1, chlorophytes, as well as the lycophyte *Selaginella moellendorffii* showed a single UVR8 copy in their genomes. However, the bryophyte *Physcomitrella patens* contains two copies located at chromosomes 3 and 10 (Supplementary Figure S1). Similarly, 41% of monocots species analyzed also showed two UVR8 copies located at different chromosomes. In the case of dicots 32% of the species analyzed contain more than one UVR8 copies in their genomes: 67% has 2 copies, 26.5% 3 copies and 6.5% has 11 copies (Supplementary Figure S1). According to the levels of overall amino acid sequence similarity, most UVR8 copies from the same species are highly related to each other showing a high level of sequence identity. It will be interesting to explore if the multiple copies of the UVR8 gene behave as a gene family and have redundant roles.

## Which is the UVR8 Ancestral Gene?

Because of the importance of “GWRHT” and “VP” domains in UVR8 function, we consider a protein as an *At*UVR8 homolog if it contains both motifs. In order to identify the UVR8 ancestral gene, we performed a PSI-BLASTp analysis against the entire NCBI database using *At*UVR8 as template. Results obtained revealed proteins with an identity below 40% from oomycetes, diatoms, and animals. The analysis of these sequences show that oomycetes conserved seven and diatoms conserved six/seven of the 14 *At*UVR8 tryptophans. Additionally, the “GWRHT” motifs that include W337 were present as “GWSHT” in oomycetes and “GWSHS” in diatoms (**Figure 2**). In addition, none of these sequences contain the “VP” core from the C27 domain (**Figure 2**). Since W337 contributes to UV-B perception but it is not essential (O’Hara and Jenkins, 2012), the presence of this motif in oomycetes and diatoms may not play a role in UV-B perception. We also performed a BLASTp analysis of Prasinophytes, Rhodophytes, Brown algae, Rhizaria, Alveolata, Excavate, Amoebozoa, Fungi, and Metazoa, which revealed the presence of proteins with identity to *At*UVR8 ranging from 29 to 36% (result not shown). Most of them were identified as putative regulators of chromosome condensation 1 or E3 ubiquitin ligase, without “GWRHT” motifs critical for UV-B perception.

The common ancestor Archaeplastida, diverged to originate three major photosynthetic groups: *Viridiplantae* (streptophyte, prasinophyte, and chlorophyte algae, as well as land plants), *Rhodophyta* (red algae), and *Glaucophyta* algae (Duanmu et al., 2014) (**Figure 1**). Several Rhodophytes organisms have sequenced genomes such as *Porphyridium purpureum, Pyropiayezoensis, Chondrus crispus, Cyanidioschyzon merolae* and *Galdieria sulphuraria.* Nonetheless, PSI-Blastp analysis revealed no *At*UVR8 homologs in any of these species, suggesting the absence of this gene in *Rhodophytes* (results no shown). This result and the absence of an UVR8 homolog in *Glaucophyta* strongly suggest that this photoreceptor was originated in the *Viridiplantae* group, specifically in the green algae lineage.

## Conclusion

Results obtained here demonstrate a functional conservation among UVR8 proteins from green algae to higher plants. The ability of different plant species to respond to UV-B determines their tolerance or sensitiveness to irradiation. The understanding of these mechanisms may improve our ability to cope with the potential effects of solar UV-B radiation on important crop yields.

## Author Contributions

MF performed bioinformatic analysis, interpreted data, drew figures, and collaborated in writing the manuscript. VT contributed to the design and analysis of the work. RC conceived the project and wrote the paper. LL supervised and improved the manuscript.

## Conflict of Interest Statement

The authors declare that the research was conducted in the absence of any commercial or financial relationships that could be construed as a potential conflict of interest.

## Footnotes

1 http://mafft.cbrc.jp/alignment/server/
