# Supplementary material for: A Comprehensive Phylogeny Reveals Functional Conservation of the UV-B Photoreceptor UVR8 from Green Algae to Higher Plants
Source: Front Plant Sci. 2016 Nov 15;7:1698. doi: 10.3389/fpls.2016.01698 (PMC5108777; doi:10.3389/fpls.2016.01698)
Supplement: Supplementary file 1 [file Data_Sheet_1.docx]

Supplementary Material

Article Title

**Authors: María Belén Fernández^1^, Vanesa Tossi^1^, Lorenzo Lamattina^1^ and Raúl Cassia^1^***

*Correspondence: Raúl Cassia: email: [rocassia@mdp.edu.ar](mailto:rocassia@mdp.edu.ar)

## Supplementary Figures


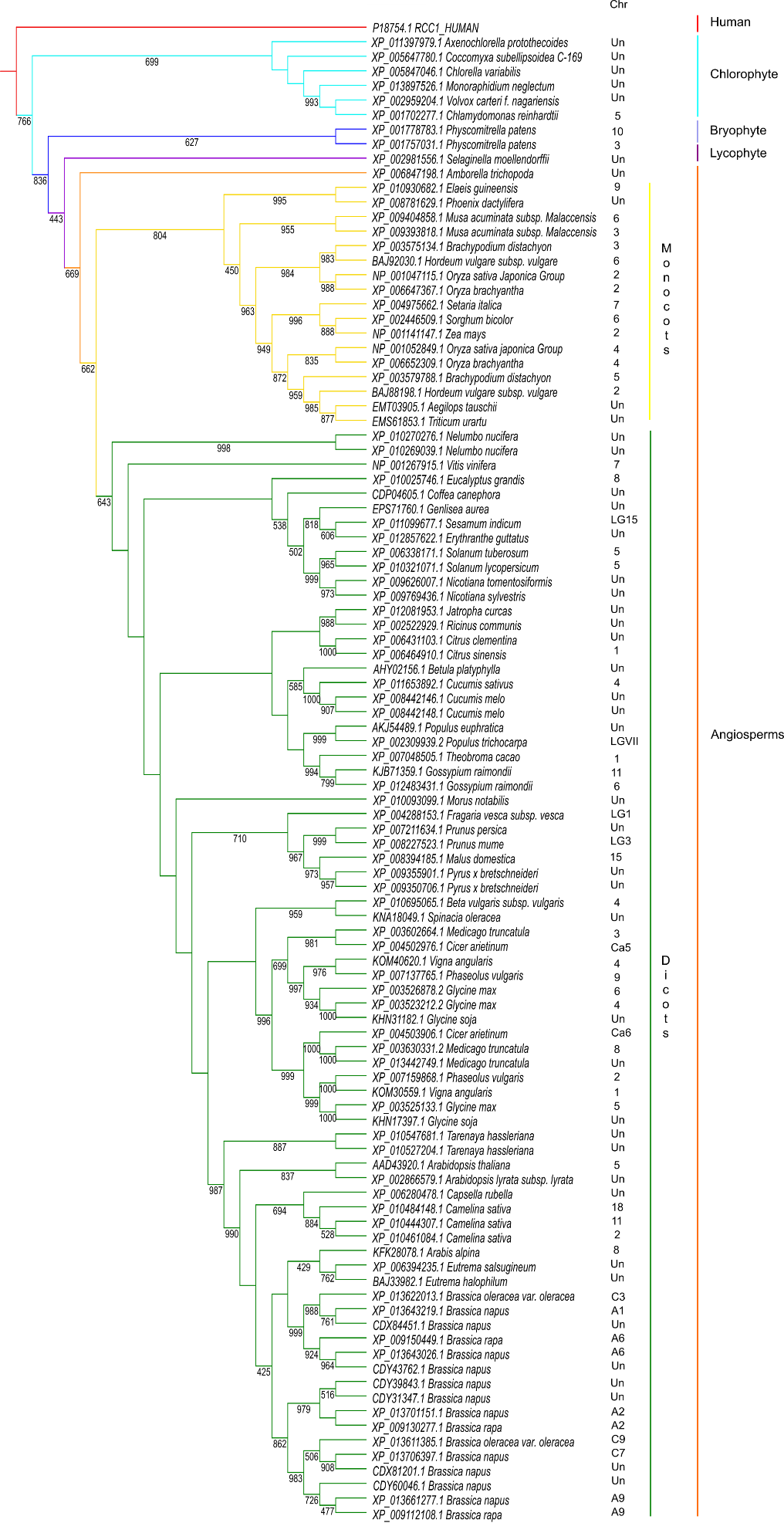


Figure S1. Phylogenetic tree of UVR8 sequences. Protein sequences with similarity to *A. thaliana* UVR8 (E-value < 9.10 e−105) were retrieved using PSI-BLAST and the non-redundant database from the *Viridiplantae* database. CDHIT software ([Huang et al., 2010](http://www.plantcell.org/content/22/11/3816.full#ref-36)) was used to remove all sequences sharing 100% identity. The sequences were aligned with MAFFT (<http://mafft.cbrc.jp/alignment/server/>) and edited with GeneDoc software (Nicholas and Nicholas, 1997). Selection of phylogenetic informative regions from the multiple sequence alignment was performed using the BMGE 1.12 software (Criscuolo and Gribaldo, 2010). Maximum likelihood phylogenetic analysis was performed with PHYML 3.0 using the JTT+G6 model (Guindon et al., 2010).Nonparametric bootstrapping (1000 replicates) was used to assess tree branching support. Bootstrap values below 40% are not shown. The program iTol (Letunic and Bork, 2016) was used to display phylogenetic trees (<http://itol.embl.de/>) and Inkscape software (https://inkscape.org/es/) for edition. Colors indicate main taxonomic divisions: Homo sapiens (red), chlorophyte (light blue), bryophyte (blue), lycophyte (violet), monocots (yellow) and dicots (green) demonstrating that each division is well resolved phylogenetically (bootstrap values > 44.3%). The accession number of each sequence is given next to the species name.


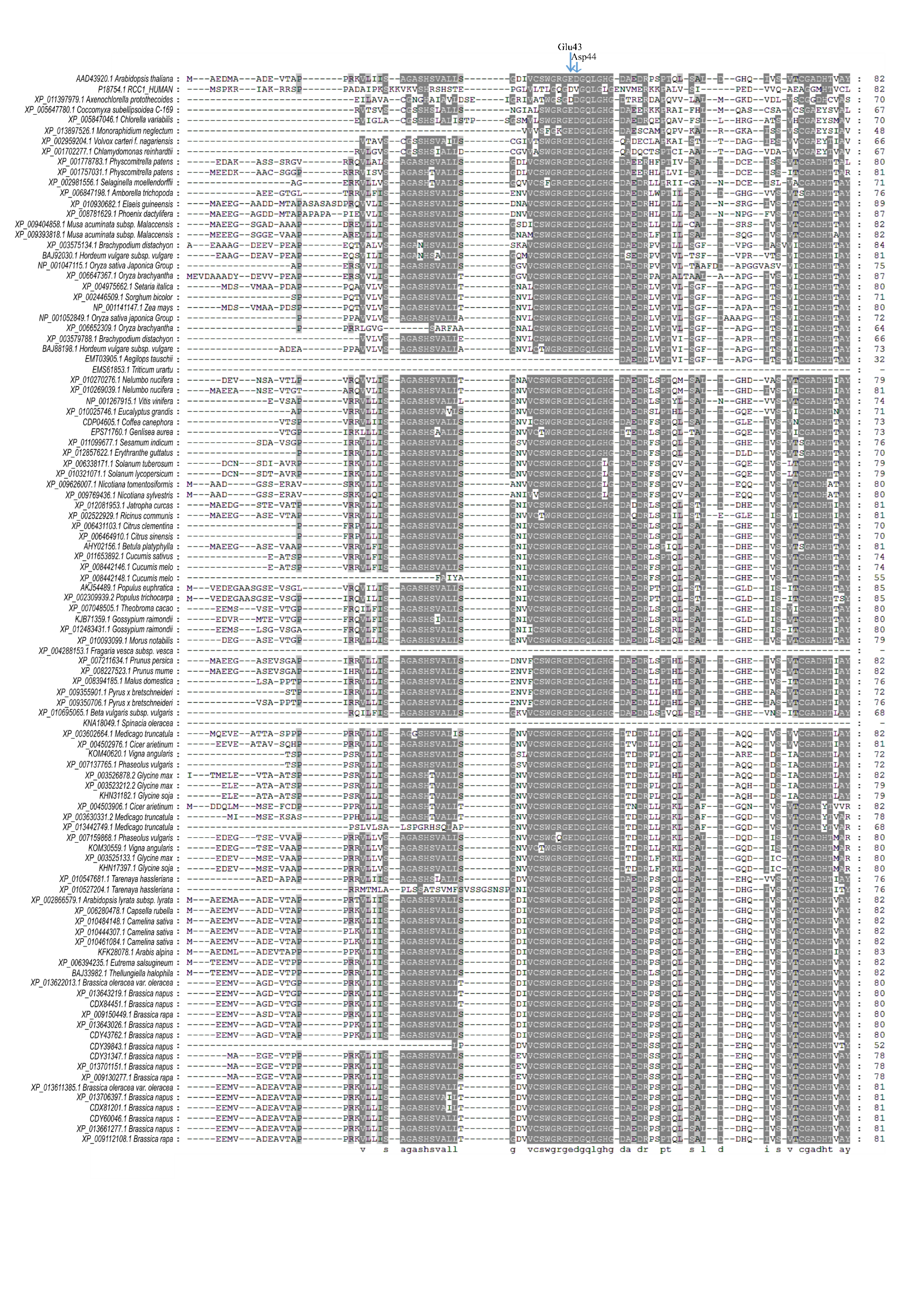


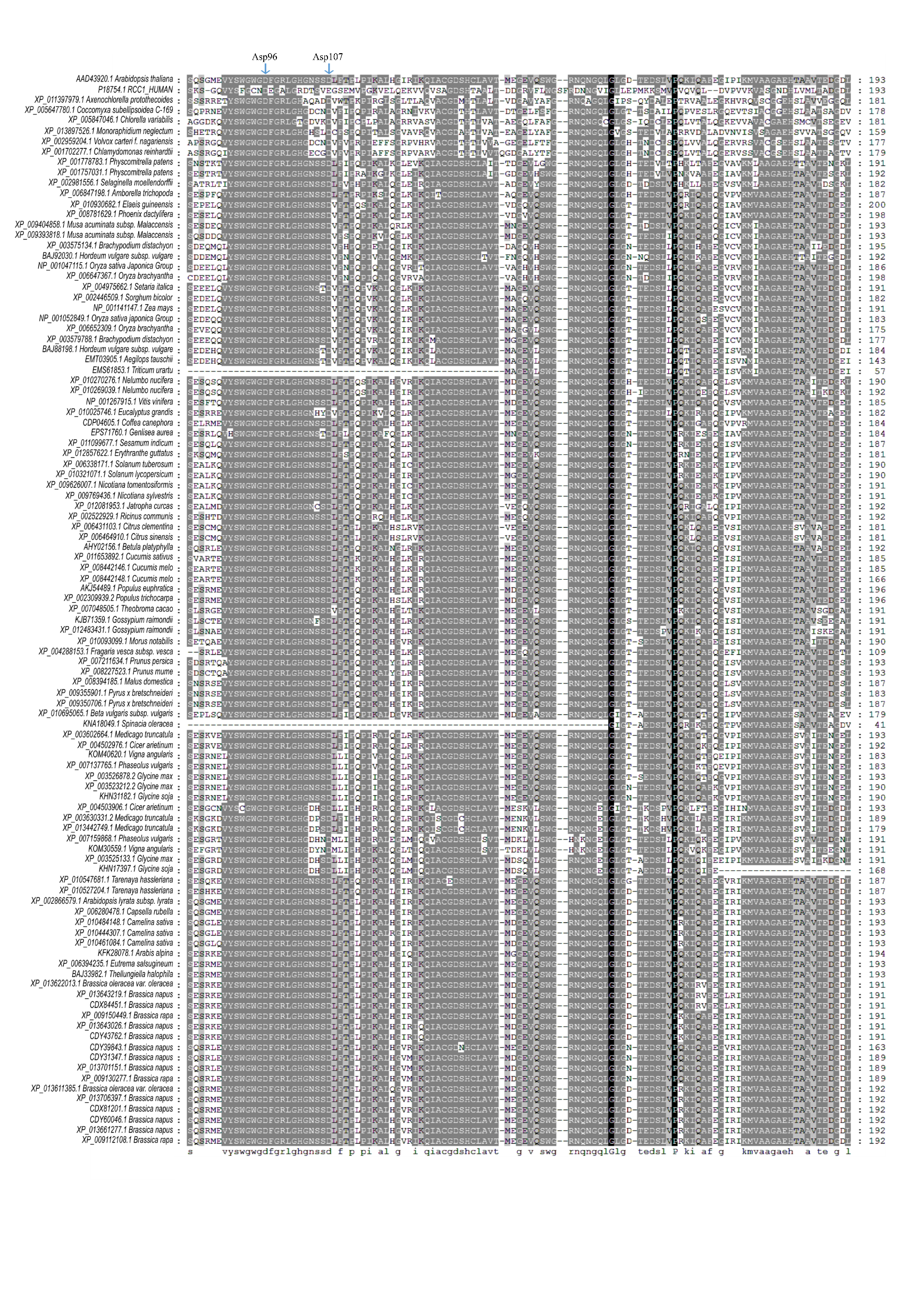


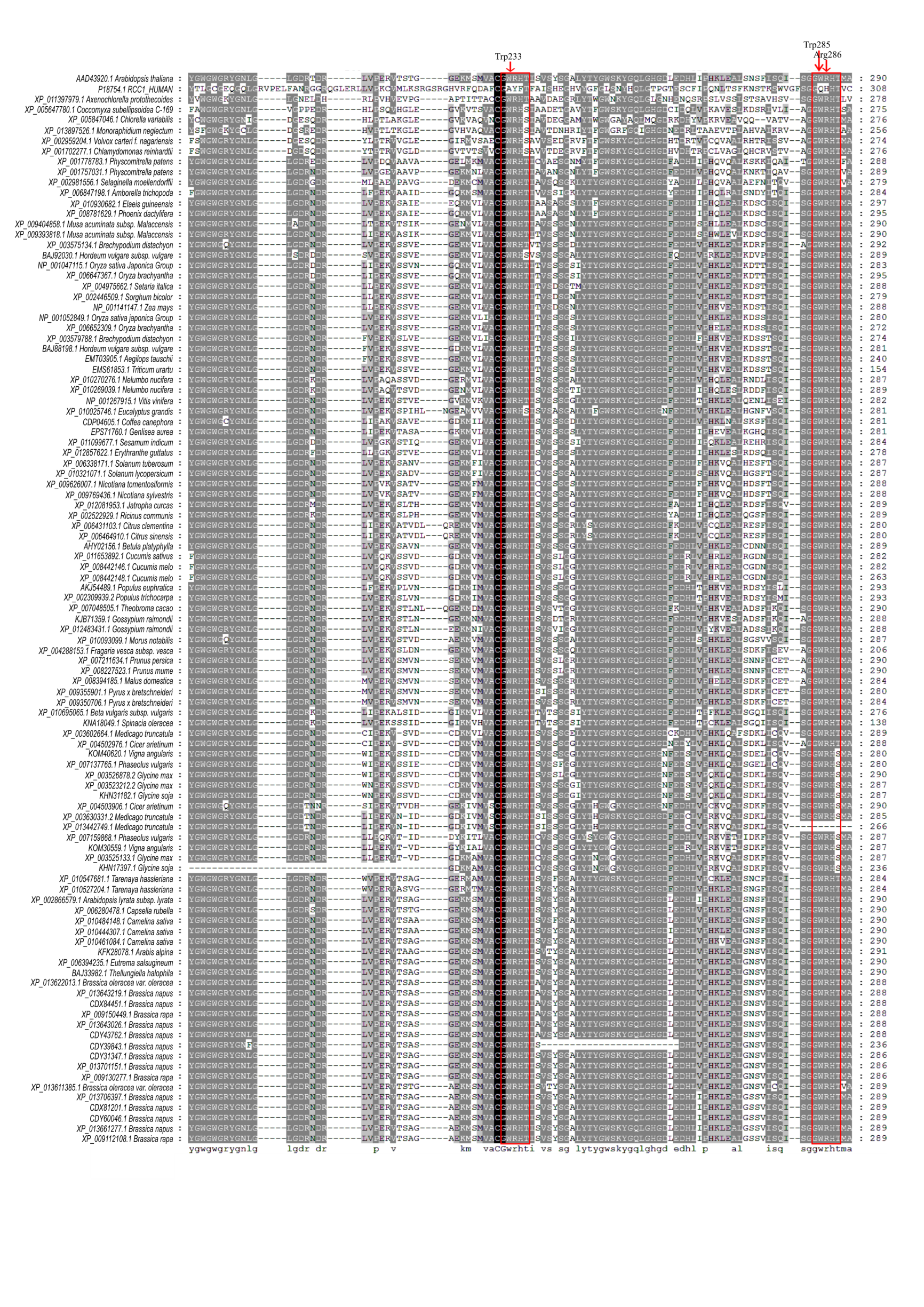


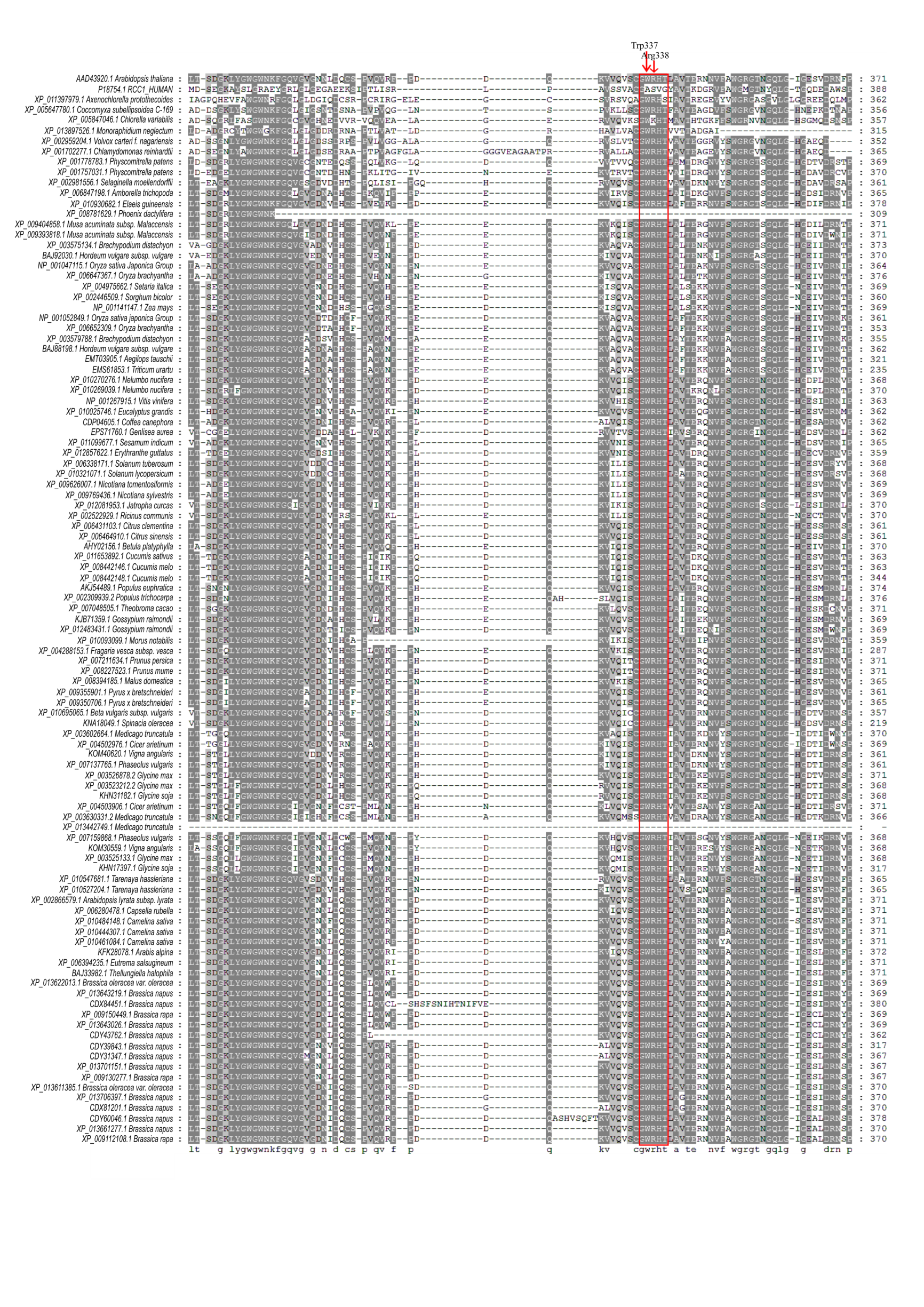


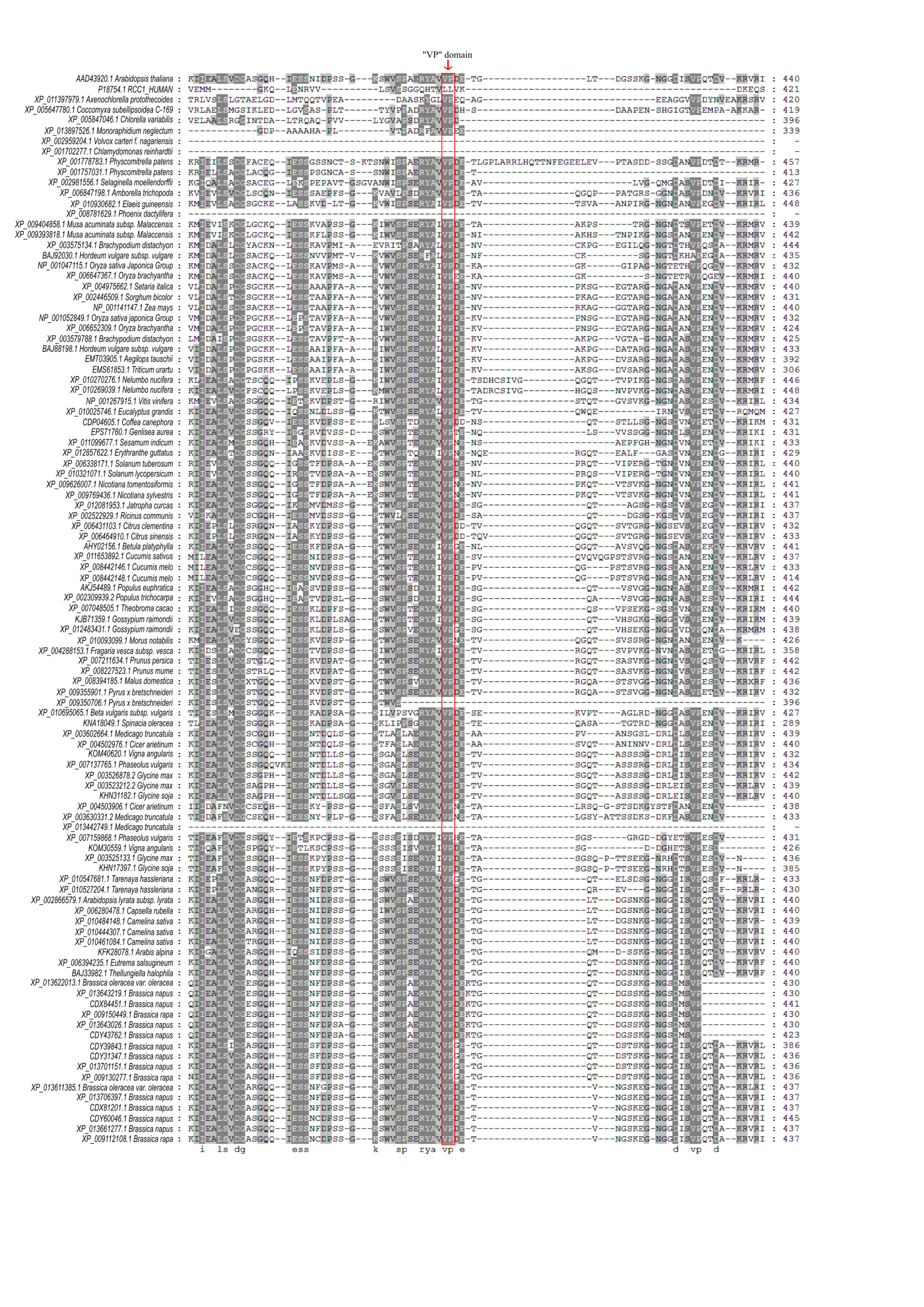


Figure S2. Multiple sequence alignment of *At*UVR8 and *Viridiplantae* homologs. Protein sequences with similarity to *A. thaliana* UVR8 were retrieved using PSI- BLASTp against the *Viridiplantae* database. The sequences were aligned with MAFFT (http://mafft.cbrc.jp/alignment/server/) and edited with GeneDoc (Nicholas and Nicholas, 1997) and Inkscape software (https://inkscape.org/es/). Boxes indicate “GWRHT” motifs and the VP domain. The accession number of each sequence is given next to the species name. Conserved residues common to all sequences are shadowed in black and less identity is shown in gray scale. Arrows indicate Glu43 and Asp44 respectively in the Arabidopsis sequence.

References

Criscuolo, A. and Gribaldo, S. (2010). BMGE (Block Mapping and Gathering with Entropy): a new software for selection of phylogenetic informative regions from multiple sequence alignments. *BMC Evol. Biol.* 10, 210. doi: 10.1186/1471-2148-10-210

[Huang, Y., Niu, B., Gao, Y., Fu, L., Li, W. (2010](http://www.plantcell.org/content/22/11/3816.full#ref-36)). CD-HIT Suite: a web server for clustering and comparing biological sequences. *Bioinformatics* 26, 680-682. doi:  [10.1093/bioinformatics/btq003](https://dx.doi.org/10.1093%2Fbioinformatics%2Fbtq003)

Letunic, I. and Bork, P. (2016). Interactive tree of life (iTOL) v3: an online tool for the display and annotation of phylogenetic and other trees. *Nucleic Acids. Res.* 44, 242-5. doi: 10.1093/nar/gkw290

Nicholas, K.B. and Nicholas, H.B.J. (1997). GeneDoc: a tool for editing and annotating multiple sequence alignments (Version 2.6.003).
